# Abdominal lymph node size in children at computed tomography

**DOI:** 10.1007/s00247-020-04715-z

**Published:** 2020-06-07

**Authors:** Suzanne Spijkers, Judith M. Staats, Annemieke S. Littooij, Rutger A. J. Nievelstein

**Affiliations:** 1 Department of Radiology and Nuclear Medicine, University Medical Centre Utrecht/Wilhelmina Children’s Hospital, Utrecht University, Heidelberglaan 100, 3584 CX Utrecht, The Netherlands; 2 Princess Máxima Centre for Paediatric Oncology, Utrecht, The Netherlands

**Keywords:** Abdomen, Child, Computed tomography, Lymph nodes, Lymphadenopathy, Reference values

## Abstract

**Background:**

Lymph node enlargement is commonly used to indicate abnormality.

**Objective:**

To evaluate the normal size and prevalence of abdominal lymph nodes in children at CT.

**Materials and methods:**

In this retrospective study, we included a total of 152 children ages 1–17 years who underwent abdominal CT examination after high-energy trauma. We measured abdominal lymph nodes in five lymph node stations (inguinal, iliac, para-aortic, hepatic and mesenteric). For the largest lymph node in each level, we measured long- and short-axis diameters in both the axial and coronal planes. We then calculated distribution parameters, correlation coefficients between lymph node size and age, and reference intervals.

**Results:**

The prevalence of detectable lymph nodes was high for the inguinal (100%), iliac (98%), para-aortic (97%) and mesenteric (99%) stations and lower for the hepatic station (32%). Lymph node size showed small to medium significant correlations (ranging from 0.21 to 0.50) with age. When applying the Lugano criteria and RECIST (Response Criteria in Solid Tumors), 29 children (19%) would have had one or more enlarged abdominal lymph nodes.

**Conclusion:**

The results of this study provide normative data of abdominal lymph node size in children. The current adult guidelines for enlarged lymph nodes seem adequate for most children with the exception of young adolescents, in which larger lymph nodes were relatively common, particularly in the inguinal region.

## Introduction

Lymph nodes in children can be enlarged for a variety of reasons, amongst which are both malignant and infectious diseases [1]. Lymph node size is often determined by US imaging, CT or MRI [2]. To correctly identify enlarged lymph nodes, it is important to know normal short- and long-axis diameter sizes for various ages and lymph node stations. Until now, no specific guidelines for evaluating abdominal lymph nodes in children have been published.

The Lugano criteria for lymphomas and Response Criteria in Solid Tumors (RECIST) are frequently used guidelines to evaluate lymph node size. The recommended cut-off values for all lymph nodes, irrespective of location and age, are 10 mm for the short axial axis diameter (both guidelines) and 15 mm for the long axial axis diameter [3, 4]. In daily clinical practice, these criteria for adults are frequently used to assess abdominal lymph nodes in children as well as in adults. During infancy, body size changes continuously and, therefore, lymph node size might change during childhood. Normal measurements of abdominal lymph nodes could thus be different for each age. In the literature, both cervical and thoracic lymph node measurements in children have been reported [5, 6], but no normative data on abdominal lymph node size in children are available.

The aim of this study was therefore to provide normative data on abdominal lymph node size in children at CT performed to rule out abdominal injuries after high-energy trauma and to assess whether abdominal lymph node size and age are correlated.

## Materials and methods

### Study population

In this retrospective study, we included children ages 1–17 years who underwent contrast-enhanced CT of the abdomen after high-energy trauma between 2012 and 2014 at the University Medical Centre Utrecht. Excluded were all abdominal CT examinations in which no intravenous contrast medium was used, the CT was performed after emergency abdominal surgery, active abdominal bleeding was visible, the CT was performed because of sharp abdominal trauma (i.e. open wounds in the abdomen) and there were any underlying diseases that could affect lymph node size (e.g., malignancies, infections). Our institutional review board gave ethics approval and waived informed consent for this retrospective study.

### Computed tomography technique

The CT images were obtained with 16×0.75-mm collimation (Mx8000 IDT or Brilliance 16P), 64×0.625-mm collimation (Brilliance 64) or 128×0.625-mm collimation (Brilliance iCT), all from Philips Medical Systems (Cleveland, OH). The exposure settings were adjusted to patient size and ranged 80–120 kV and 21–307 mAs. The reconstructed axial images had a slice-thickness of 5.0 mm and the coronal images were 3.0 mm. All CT scans were contrast-enhanced following the local protocol; each child received 2 mL/kg body weight contrast agent with a maximum of 125 mL (body weight <85 kg) or a maximum of 150 mL (body weight >85 kg).

### Computed tomography measurements

All measurements were performed by one observer (J.M.S., medical student). To determine interobserver agreement, a subset of 20 CT scans was separately evaluated by an experienced paediatric radiologist (R.A.J.N., with 25 years of experience in paediatric abdominal CT). The sample used for assessing interobserver agreement was taken randomly from all examined CTs with the premises that every age was represented. In the following lymph node stations the largest lymph node was measured [7]:

- Inguinal: lymph nodes located below the inguinal ligament,
- Iliac: lymph nodes located between the aortic bifurcation and the inguinal ligament,
- Hepatic: lymph nodes situated along the course of the hepatic artery,
- Para-aortic: lymph nodes located directly around the abdominal aorta, both above and below the renal hilus; and
- Mesenteric: lymph nodes situated along the course of the mesenteric vessels.

Four measurements were performed per lymph node: the short- and long-axis diameters in the axial and coronal planes.

### Statistical analysis

We analysed lymph node measurements per age and lymph node station, calculating the median, interquartile range (IQR) and maximum sizes of the lymph nodes per axis, age and lymph node station. Because of the non-normal distribution of the data, we calculated Pearson correlation coefficients (including *P*-values and 95% confidence intervals [CIs]) between age and lymph node size using bootstrapping with bias-corrected and accelerated confidence estimates with 5,000 bootstrap resamples. To generate more generalizable reference values and to be able to compare our data to the current RECIST and Lugano guidelines, we calculated mean values and upper limits of the reference interval per axis and lymph node station [3, 4]. The reference limits and confidence intervals were estimated using bootstrapping with robust methods. For the comparison of the long axial and the long coronal axes, we used a linear mixed model to account for clustering within patients. To assess both the interobserver variability and intraobserver variability, we used Bland–Altman plots. For all analyses, we used the Statistical Package for the Social Sciences (SPSS) version 25.0 for Windows (IBM, Armonk, NY) and the R statistical software package (R Development Core Team, Vienna, Austria). *P*-values <0.05 were considered statistically significant.

## Results

**Fig. 1:**
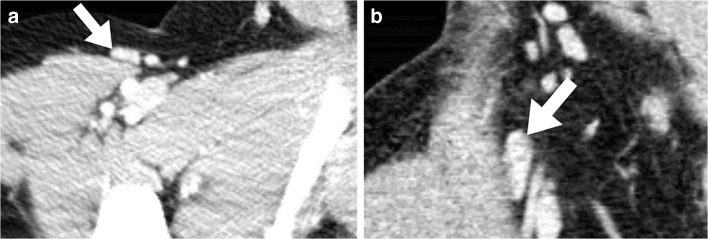
Abdominal contrast-enhanced CT images (120 kV, 50 mAs) in a 15-year-old boy. **a** Axial reconstruction shows an inguinal lymph node (*arrow*) with a short-axis diameter of 7 mm and a long-axis diameter of 15 mm. **b** Corresponding image in the coronal plane shows the same inguinal lymph node (*arrow*) with a short-axis diameter of 12 mm and a long-axis diameter of 30 mm

A total of 154 children were retrospectively selected for inclusion, of whom 2 children were excluded before analyses because the CT was performed after (emergency) abdominal surgery. Therefore, a total of 152 children, 96 boys (63%) and 56 girls (37%), were included in this study. For every child, we measured lymph nodes in at least 3 abdominal lymph node stations for a total of 647 lymph nodes. Inguinal lymph nodes were found in 152 children (100%), iliac lymph nodes in 149 children (98%), para-aortic lymph nodes in 148 children (97%), hepatic hilar lymph nodes in 48 children (32%), and mesenteric lymph nodes in 150 children (99%). In Tables 1, 2, 3 and 4 we summarize the prevalence, median size, IQR and maximum size of the lymph nodes for all four measured axes (both the short and long axes in the axial and coronal planes), per age and lymph node station. In 68% of all measured lymph nodes (440/647) the long coronal axis exceeded the long axial axis, indicating a vertical orientation of the lymph node. The long axes in the axial and coronal planes differed significantly, with a *P*-value of <0.01. Figure 1 shows an example of a vertically orientated inguinal lymph node.

**Table 1:** Axial plane, short axis: size (in millimeters) and prevalence of abdominal lymph nodes on multidetector CT in children (*n*)

Age (years)	*n*	Inguinal			Iliac			Para–aortic			Hepatic			Mesenteric		
		*n* (%)	Median (IQR)	Max	*n* (%)	Median (IQR)	Max	*n* (%)	Median (IQR)	Max	*n* (%)	Median (IQR)	Max	*n* (%)	Median (IQR)	Max
1	6	6 (100)	6 (5–7)	8	5 (83)	4 (3–4)	4	6 (100)	4 (3–4)	5	0 (0)	–	–	4 (67)	4 (3–6)	7
2	7	7 (100)	5 (5–6)	7	7 (100)	4 (3–4)	5	7 (100)	4 (3–6)	7	0 (0)	–	–	7 (100)	7 (5–8)	8
3	6	6 (100)	6 (4–6)	6	6 (100)	4 (3–5)	5	5 (83)	3 (3–4)	5	0 (0)	–	–	6 (100)	6 (5–7)	7
4	5	5 (100)	6 (5–6)	6	5 (100)	4 (4–5)	5	5 (100)	3 (3–4)	4	1 (20)	3	3	5 (100)	6 (5–8)	8
5	6	6 (100)	8 (7–8)	9	6 (100)	6 (5–6)	6	6 (100)	4 (3–5)	6	2 (33)	3 (3–3)	3	6 (100)	6 (5–8)	9
6	6	6 (100)	6 (5–7)	8	5 (83)	4 (4–5)	5	5 (83)	4 (4–6)	6	1 (17)	3	3	6 (100)	5 (4–8)	9
7	5	5 (100)	6 (6–7)	7	5 (100)	4 (4–6)	6	5 (100)	5 (4–7)	7	1 (20)	6	6	5 (100)	6 (6–8)	8
8	7	7 (100)	5 (5–7)	8	7 (100)	5 (5–5)	6	7 (100)	4 (3–5)	6	3 (43)	5 (4–5)	5	7 (100)	5 (4–6)	7
9	5	5 (100)	5 (5–7)	7	5 (100)	5 (5–7)	8	5 (100)	4 (3–5)	6	1 (20)	5	5	5 (100)	6 (5–7)	7
10	7	7 (100)	6 (4–7)	8	7 (100)	5 (4–7)	7	7 (100)	4 (4–5)	6	3 (43)	3 (3–4)	4	7 (100)	6 (5–7)	8
11	6	6 (100)	7 (6–8)	8	6 (100)	4 (4–6)	7	6 (100)	5 (5–5)	5	3 (50)	6 (3–6)	6	6 (100)	6 (5–6)	7
12	10	10 (100)	7 (5–8)	10	10 (100)	6 (5–7)	9	10 (100)	5 (5–6)	6	4 (40)	5 (4–7)	7	10 (100)	6 (5–8)	9
13	9	9 (100)	6 (5–7)	9	9 (100)	5 (4–7)	9	7 (78)	4 (4–6)	7	0 (0)	–	–	9 (100)	5 (5–6)	8
14	10	10 (100)	7 (6–8)	10	10 (100)	5 (5–6)	7	10 (100)	5 (4–5)	6	4 (40)	6 (5–8)	8	10 (100)	6 (5–7)	9
15	14	14 (100)	8 (6–8)	9	14 (100)	7 (5–7)	8	14 (100)	5 (4–5)	8	8 (57)	5 (4–6)	7	14 (100)	7 (6–7)	10
16	16	16 (100)	8 (6–9)	12	15 (94)	5 (5–8)	11	16 (100)	5 (4–6)	9	8 (50)	4 (3–5)	8	16 (100)	6 (4–7)	12
17	27	27 (100)	7 (7–9)	14	27 (100)	6 (5–6)	9	27 (100)	5 (4–5)	6	9 (33)	5 (5–7)	7	27 (100)	6 (5–7)	9

*IQR* interquartile range, *Max* maximum

**Table 2:** Axial plane, long axis: size (in millimeters) and prevalence of abdominal lymph nodes on multidetector CT in children (*n*)

Age (years)	*n*	Inguinal			Iliac			Para–aortic			Hepatic			Mesenteric		
		*n* (%)	Median (IQR)	Max	*n* (%)	Median (IQR)	Max	*n* (%)	Median (IQR)	Max	*n* (%)	Median (IQR)	Max	*n* (%)	Median (IQR)	Max
1	6	6 (100)	10 (9–12)	14	5 (83)	5 (4–6)	6	6 (100)	5 (3–7)	8	0 (0)	–	–	4 (67)	7 (5–10)	11
2	7	7 (100)	10 (9–10)	11	7 (100)	6 (4–8)	8	7 (100)	7 (6–9)	14	0 (0)	–	–	7 (100)	11 (8–15)	15
3	6	6 (100)	9 (7–10)	12	6 (100)	6 (5–6)	7	5 (83)	4 (4–6)	6	0 (0)	–	–	6 (100)	9 (7–11)	11
4	5	5 (100)	9 (8–10)	10	5 (100)	7 (6–7)	7	5 (100)	6 (4–6)	6	1 (20)	4	4	5 (100)	8 (8–11)	12
5	6	6 (100)	12 (10–14)	14	6 (100)	8 (7–8)	9	6 (100)	6 (5–7)	8	2 (33)	5 (4–5)	5	6 (100)	9 (7–12)	12
6	6	6 (100)	12 (10–13)	14	5 (83)	7 (5–8)	8	5 (83)	6 (5–7)	7	1 (17)	5	5	6 (100)	8 (6–13)	14
7	5	5 (100)	9 (8–11)	12	5 (100)	7 (6–10)	11	5 (100)	7 (6–9)	10	1(20)	8	8	5 (100)	9 (8–12)	12
8	7	7 (100)	10 (9–12)	16	7 (100)	8 (7–11)	12	7 (100)	5 (5–7)	11	3 (43)	5 (5–8)	8	7 (100)	8 (6–11)	12
9	5	5 (100)	7 (7–12)	13	5 (100)	7 (6–11)	11	5 (100)	5 (5–8)	10	1 (20)	6	6	5 (100)	9 (9–11)	11
10	7	7 (100)	11 (8–13)	14	7 (100)	8 (6–12)	12	7 (100)	7 (5–8)	8	3 (43)	6 (5–6)	6	7 (100)	8 (6–10)	10
11	6	6 (100)	12 (12–15)	15	6 (100)	8 (6–11)	12	6 (100)	8 (8–8)	9	3 (50)	8 (4–9)	9	6 (100)	9 (7–9)	10
12	10	10 (100)	11 (10–13)	16	10 (100)	8 (7–10)	11	10 (100)	8 (6–10)	11	4 (40)	8 (6–12)	13	10 (100)	10 (9–11)	13
13	9	9 (100)	8 (8–9)	13	9 (100)	7 (7–12)	15	7 (78)	6 (6–7)	8	0 (0)	–	–	9 (100)	9 (7–12)	14
14	10	10 (100)	10 (9–12)	12	10 (100)	9 (8–10)	11	10 (100)	7 (5–8)	8	4 (40)	10 (7–12)	12	10 (100)	9 (7–10)	13
15	14	14 (100)	11 (10–14)	15	14 (100)	9 (8–12)	16	14 (100)	7 (6–8)	10	8 (57)	7 (6–9)	12	14 (100)	12 (11–13)	15
16	16	16 (100)	12 (11–13)	17	15 (94)	8 (7–10)	17	16 (100)	8 (6–8)	13	8 (50)	6 (5–9)	15	16 (100)	11 (7–12)	19
17	27	27 (100)	12 (10–15)	17	27 (100)	9 (7–12)	17	27 (100)	7 (6–8)	11	9 (33)	7 (6–9)	11	27 (100)	10 (8–13)	19

*IQR* interquartile range, *Max* maximum

**Table 3:** Coronal plane, short axis: size (in millimeters) and prevalence of abdominal lymph nodes on multidetector CT in children (*n*)

Age (years)	*n*	Inguinal			Iliac			Para–aortic			Hepatic			Mesenteric		
		*n* (%)	Median (IQR)	Max	*n* (%)	Median (IQR)	Max	*n* (%)	Median (IQR)	Max	*n* (%)	Median (IQR)	Max	*n* (%)	Median (IQR)	Max
1	6	6 (100)	8 (5–10)	10	5 (83)	5 (3–6)	6	6 (100)	4 (3–4)	5	0 (0)	–	–	4 (67)	4 (3–8)	9
2	7	7 (100)	6 (6–7)	8	7 (100)	5 (4–5)	5	7 (100)	4 (3–5)	5	0 (0)	–	–	7 (100)	8 (7–11)	12
3	6	6 (100)	7 (6–7)	8	6 (100)	4 (4–6)	7	5 (83)	3 (3–4)	4	0 (0)	–	–	6 (100)	7 (5–8)	9
4	5	5 (100)	7 (5–8)	8	5 (100)	6 (5–6)	6	5 (100)	4 (3–4)	4	1 (20)	4	4	5 (100)	6 (6–8)	8
5	6	6 (100)	9 (7–10)	10	6 (100)	5 (4–5)	5	6 (100)	4 (4–4)	5	2 (33)	3 (2–3)	3	6 (100)	7 (5–10)	10
6	6	6 (100)	7 (5–9)	10	5 (83)	6 (5–6)	6	5 (83)	5 (4–6)	6	1 (17)	6	6	6 (100)	6 (5–9)	9
7	5	5 (100)	8 (6–10)	11	5 (100)	5 (5–6)	6	5 (100)	4 (4–5)	5	1(20)	4	4	5 (100)	8 (6–11)	12
8	7	7 (100)	7 (7–8)	9	7 (100)	6 (4–7)	8	7 (100)	6 (4–8)	8	3 (43)	4 (4–6)	6	7 (100)	6 (5–8)	8
9	5	5 (100)	6 (5–8)	8	5 (100)	6 (5–7)	7	5 (100)	3 (3–5)	6	1 (20)	3	3	5 (100)	6 (6–9)	9
10	7	7 (100)	6 (6–10)	11	7 (100)	6 (4–8)	8	7 (100)	5 (4–6)	6	3 (43)	4 (3–4)	5	7 (100)	5 (5–7)	8
11	6	6 (100)	10 (8–11)	11	6 (100)	6 (5–9)	10	6 (100)	5 (4–5)	6	3 (50)	4 (4–6)	6	6 (100)	7 (5–7)	8
12	10	10 (100)	8 (8–10)	14	10 (100)	6 (5–7)	9	10 (100)	5 (4–6)	8	4 (40)	4 (3–6)	6	10 (100)	9 (7–10)	12
13	9	9 (100)	7 (7–8)	11	9 (100)	6 (5–9)	13	7 (78)	4 (4–5)	5	0 (0)	–	–	9 (100)	7 (5–10)	12
14	10	10 (100)	8 (6–9)	11	10 (100)	6 (5–7)	7	10 (100)	4 (3–6)	7	4 (40)	4 (3–8)	9	10 (100)	9 (6–9)	10
15	14	14 (100)	9 (8–11)	12	14 (100)	7 (6–9)	12	14 (100)	6 (5–7)	8	8 (57)	5 (4–6)	9	14 (100)	7 (6–9)	11
16	16	16 (100)	8 (7–10)	14	15 (94)	5 (5–7)	12	16 (100)	5 (4–7)	9	8 (50)	4 (3–5)	10	16 (100)	7 (6–8)	11
17	27	27 (100)	9 (7–11)	15	27 (100)	6 (5–8)	10	27 (100)	5 (5–6)	8	9 (33)	6 (4–7)	7	27 (100)	7 (6–9)	10

*IQR* interquartile range, *Max* maximum

**Table 4:** Coronal plane, long axis: size (in millimeters) and prevalence of abdominal lymph nodes on multidetector CT in children (*n*)

Age (yrs)	*n*	Inguinal			Iliac			Para–aortic			Hepatic			Mesenteric		
		*n* (%)	Median (IQR)	Max	*n* (%)	Median (IQR)	Max	*n* (%)	Median (IQR)	Max	*n* (%)	Median (IQR)	Max	*n* (%)	Median (IQR)	Max
1	6	6 (100)	13 (9–15)	18	5 (83)	6 (5–7)	7	6 (100)	6 (5–7)	8	0 (0)	–	–	4 (67)	8 (6–12)	13
2	7	7 (100)	12 (10–19)	19	7 (100)	7 (6–8)	8	7 (100)	7 (6–8)	8	0 (0)	–	–	7 (100)	14 (10–15)	16
3	6	6 (100)	11 (9–14)	15	6 (100)	8 (7–9)	10	5 (83)	5 (4–6)	6	0 (0)	–	–	6 (100)	10 (7–11)	12
4	5	5 (100)	13 (11–18)	18	5 (100)	8 (7–9)	9	5 (100)	5 (4–7)	8	1 (20)	5	5	4 (80)	7 (5–12)	14
5	6	6 (100)	19 (15–20)	23	6 (100)	8 (6–10)	11	6 (100)	7 (5–8)	9	2 (33)	4 (3–4)	4	5 (83)	16 (9–18)	19
6	6	6 (100)	16 (11–17)	17	5 (83)	8 (7–9)	9	5 (83)	6 (6–8)	8	1 (17)	8	8	6 (100)	11 (9–13)	16
7	5	5 (100)	13 (11–18)	20	5 (100)	9 (9–12)	14	5 (100)	6 (5–8)	9	1(20)	5	5	4 (80)	13 (10–14)	14
8	7	7 (100)	14 (11–15)	16	7 (100)	11 (10–13)	13	7 (100)	6 (5–7)	12	3 (43)	5 (4–5)	6	6 (86)	10 (8–13)	13
9	5	5 (100)	12 (10–22)	23	5 (100)	9 (6–15)	15	5 (100)	5 (4–7)	9	1 (20)	4	4	3 (60)	9 (3–9)	12
10	7	7 (100)	13 (11–14)	23	7 (100)	12 (9–19)	19	7 (100)	7 (6–8)	8	3 (43)	6 (5–6)	6	4 (57)	13 (7–14)	14
11	6	6 (100)	17 (16–17)	18	6 (100)	9 (8–12)	17	6 (100)	9 (6–11)	12	3 (50)	7 (4–9)	9	5 (83)	10 (9–11)	11
12	10	10 (100)	15 (12–17)	24	10 (100)	11 (8–16)	21	10 (100)	11 (7–11)	16	4 (40)	7 (5–12)	13	10 (100)	12 (9–14)	19
13	9	9 (100)	14 (10–17)	20	9 (100)	13 (9–17)	19	7 (78)	8 (8–10)	14	0 (0)	–	–	9 (100)	12 (11–16)	19
14	10	10 (100)	13 (9–19)	21	10 (100)	10 (9–13)	19	10 (100)	6 (5–10)	18	4 (40)	8 (6–11)	12	10 (100)	14 (11–16)	18
15	14	14 (100)	17 (15–25)	31	14 (100)	13 (9–17)	26	14 (100)	9 (6–12)	16	8 (57)	8 (6–9)	13	14 (100)	12 (10–13)	18
16	16	16 (100)	14 (12–21)	29	15 (94)	10 (7–15)	23	16 (100)	8 (6–14)	17	8 (50)	8 (6–8)	15	16 (100)	12 (10–14)	21
17	27	27 (100)	15 (13–19)	29	27 (100)	13 (9–16)	23	27 (100)	9 (7–11)	19	9 (33)	9 (7–10)	15	27 (100)	12 (10–14)	18

*IQR* interquartile range, *Max* maximum, *yrs* years

To compare the study population with current adult guidelines, Table 5 summarizes the upper limits of the reference interval per axis and per lymph node level. The upper limits of the short axial axis of all lymph node stations are quite similar to those in current adult guidelines (cut-off of <10 mm for all short-axis diameters) and range from 6.4 mm to 10.0 mm. The short coronal axis upper limits of the reference interval exceed 10 mm for the inguinal (12.4 mm) and mesenteric (11.2 mm) stations. For the long-axis diameter, the adult guidelines recommend a cut-off point of 15 mm. In the axial plane, the iliac, para-aortic, hepatic and mesenteric upper limits remain below or equal to 15 mm (10.2–15.0 mm). However, the inguinal upper limit exceeds this threshold with 15.8 mm. The long coronal axis diameter upper limit exceeded 15 mm for the inguinal, iliac and mesenteric stations (range 18.4–24.6 mm). For the para-aortic and hepatic lymph node stations, the upper limit did not exceed 15 mm (14.4 mm and 12.8 mm respectively).

**Table 5:** Means and upper limits of the reference interval including 95% confidence intervals (CI) in millimetres (mm) per axis and lymph node station

Lymph node station	Short axial axis		Long axial axis		Short coronal axis		Long coronal axis	
	Mean [95% CI]	Upper limit reference interval (95% CI)	Mean [95% CI]	Upper limit reference interval (95% CI)	Mean [95% CI]	Upper limit reference interval (95% CI)	Mean [95% CI]	Upper limit reference interval (95% CI)
Inguinal	6.7 [6.4–7.0]	10.0 [9.6–10.5]	10.9 [10.5–11.3]	15.8 [15.1–16.5]	8.2 [7.8–8.5]	12.4 [11.8–13.0]	15.2 [14.5–16.0]	24.6 [23.3–25.9]
Iliac	5.3 [5.0–5.5]	8.0 [7.6–8.4]	8.2 [7.8–8.7]	13.5 [12.8–14.2]	6.0 [5.7–6.3]	9.3 [8.8–9.7]	11.0 [10.3–11.7]	19.4 [18.3–20.6]
Para–aortic	4.6 [4.4–4.8]	6.4 [6.1–6.7]	6.8 [6.5–7.1]	10.2 [9.7–10.6]	4.7 [4.5–4.9]	6.8 [6.5–7.1]	8.0 [7.5–8.6]	14.4 [13.5–15.3]
Hepatic	4.8 [4.4–5.2]	7.5 [7.1–7.9]	7.1 [6.5–7.9]	12.1 [11.4–12.8]	4.7 [4.2–5.2]	8.0 [7.6–8.5]	7.4 [6.6–8.2]	12.8 [12.0–13.5]
Mesenteric	6.1 [5.9–6.4]	9.0 [8.6–9.4]	9.7 [9.3–10.2]	15.0 [14.3–15.8]	7.1 [6.8–7.5]	11.2 [10.6–11.8]	11.6 [11.0–12.2]	18.4 [17.4–19.3]

**Fig. 2:**
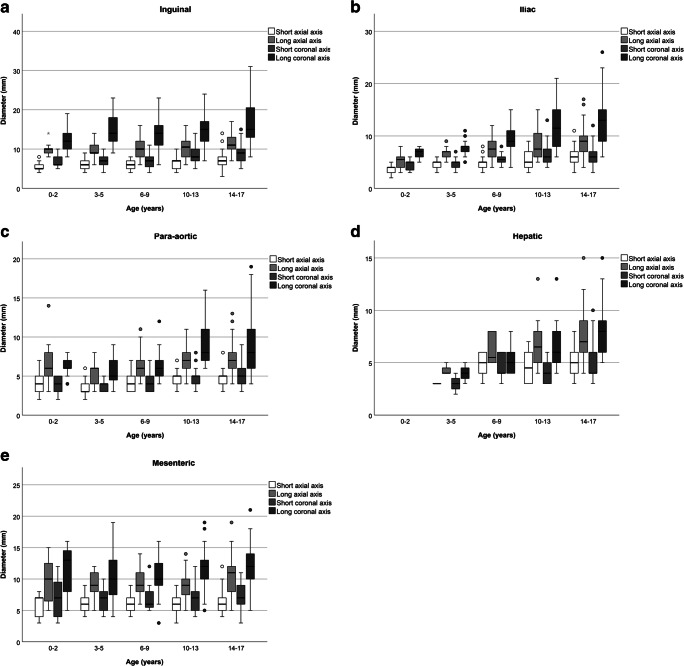
Boxplots by age group for all five abdominal lymph node stations. Boxplots represent: mean, interquartile range (IQR) and ranges of diameter sizes (mm). **a** Inguinal lymph nodes. **b** Iliac lymph nodes. **c** Paraaortic lymph nodes. **d** Hepatic lymph nodes. **e** Mesenteric lymph nodes

When applying the Lugano and RECIST criteria on the axial measurements, 29 children (19%), of whom 5 (17%) were younger than 12 years and 24 (83%) were 12 years or older, had one or more abdominal lymph nodes exceeding upper normal limits (21 [60%] inguinal, 5 [14%] iliac, 1 [3%] hepatic, 8 [23%] mesenteric). We found no para-aortic lymph nodes exceeding the Lugano and RECIST criteria. For the coronal measurements, a total of 105 children (69%) had one or more lymph nodes that exceeded the Lugano and RECIST criteria (76 [50%] inguinal, 31 [20%] iliac, 2 [1%] hepatic, 10 [7%] para-aortic, 33 [22%] mesenteric). Figure 2 shows boxplots representing the size of the abdominal lymph nodes per axis, lymph node station and age group.

Pearson correlation coefficients for age and lymph node size per axis and lymph node station are shown in Table 6. Small to medium significant correlations between age and lymph node size were found for all lymph node stations (0.21–0.50, *P*<0.05).

**Table 6:** Pearson correlation coefficients for age and lymph node size, with confidence intervals based on 5,000 bootstrap samples

Lymph node station	Short axial axis Coefficient [95% CI]	Long axial axis Coefficient [95% CI]	Short coronal axis Coefficient [95% CI]	Long coronal axis Coefficient [95% CI]
Inguinal	0.40 [0.28–0.52] (*P*<0.01)	0.30 [0.16–0.43] (*P*<0.01)	0.37 [0.23–0.48] (*P*<0.01)	0.25 [0.10–0.38] (*P*<0.01)
Iliac	0.44 [0.32–0.55] (*P*<0.01)	0.44 [0.31–0.55] (*P*<0.01)	0.38 [0.26–0.51] (*P*<0.01)	0.47 [0.36–0.59] (*P*<0.01)
Para–aortic	0.35 [0.19–0.50] (*P*<0.01)	0.23 [0.05–0.41 (*P*=0.04)	0.41 [0.30–0.50] (*P*<0.01)	0.44 [0.34–0.53] (*P*<0.01)
Hepatic	0.29 [0.02–0.51] (*P*=0.04)	0.29 [0.05–0.49] (*P*=0.04)	0.28 [0.06–0.48] (*P=*0.05)	0.50 [0.28–0.67] (*P*<0.01)
Mesenteric	0.13 [−0.03–0.28] (*P*=0.09)	0.21 [0.03–0.37] (*P*=0.01)	0.10 [−0.08–0.28] (*P=*0.20)	0.21 [0.04–0.38] (*P*=0.01)

*CI* confidence interval

**Fig. 3:**
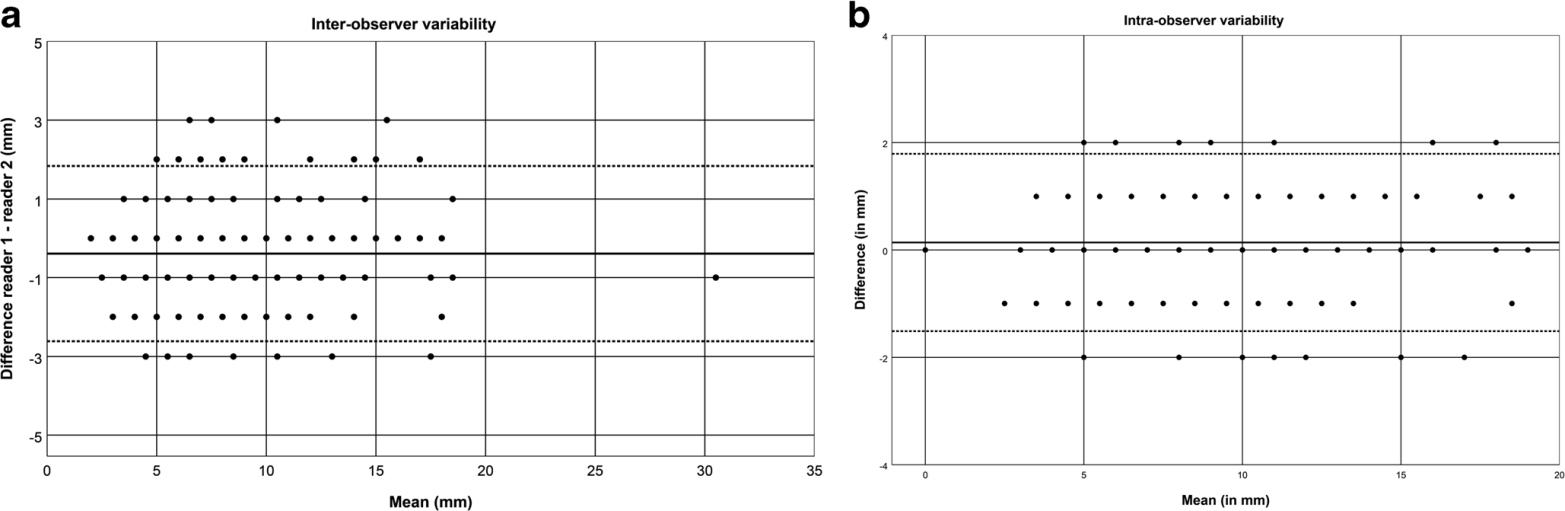
Bland–Altman plots of observer variability. The average measurements (x-axis) are plotted against their difference (y-axis). **a** Bland–Altman plot compares measurements of Reader 1 (J.M.S.) and Reader 2 (R.A.J.N.). The mean difference (−0.39 mm) is shown by the continuous line, whereas the dotted lines represent the 95% confidence intervals of the average difference in measurements (−2.61 mm to 1.83 mm). **b** Bland–Altman plot shows intraobserver variability in 20 children (reader J.M.S.). The continuous line represents the mean difference (0.14 mm), whereas the dotted lines represent the 95% confidence intervals of the average difference in measurements (−1.51 to 1.79 mm)

We assessed interobserver agreement for the measurements of 83 lymph nodes in 20 children between both readers using a Bland–Altman plot (Fig. 3). The mean difference between the measurements of both readers was –0.39 mm and the 95% limits of agreement were –2.61 mm to 1.83 mm. For the intraobserver variability in 20 children, the mean difference between readings was 0.14 mm and the 95% confidence intervals of the average difference in measurements were−1.51 mm to 1.79 mm (Fig. 3).

## Discussion

In this study we provide normative data on prevalence and size of short- and long-axis diameters in both the axial and coronal planes of abdominal lymph nodes on abdominal CT in children ages 1–17 years. The prevalence of abdominal lymph nodes in children was high in the inguinal, iliac, mesenteric and para-aortic lymph node stations, corresponding to previous studies in adults and children on CT [8–12]. The lower prevalence of hepatic lymph nodes found in this study (32%) probably does not indicate a general absence of hepatic lymph nodes but is most likely related to a lesser visibility of lymph nodes due to a scarcity of intra-abdominal fat around the liver because in clinical practice hepatic lymph nodes are frequently found in children on US imaging. This is supported by a former study with less advanced CT techniques, describing an even lower prevalence in healthy adults (10%) [8].

When applying the Lugano and RECIST criteria to the study population, in 19% of the children one or more enlarged abdominal lymph nodes were found. The clinical implication of this result is quite large: nearly one-fifth of the healthy children in this cohort would have required additional examination, causing unnecessary stress and anxiety for both child and parents. Seventy-six percent of the children with enlarged lymph nodes were older than 12 years and the most commonly involved lymph node station was the inguinal station. This is not surprising because inguinal lymph nodes in clinical practice are often large and well palpable in children, which is similar to cervical lymph nodes [13]. Our results are comparable to a recent study on inguinal lymph nodes in a healthy adult population, in which they reported a long axial axis ranging 6.0–23.5 mm and a short axial axis ranging 2.1–13.6 mm [9]. Previous studies on mesenteric lymph nodes on CT reported upper limits for the short axial axis of 10 mm in children and 11 mm in adults and a long axial axis of 20 mm [10–12]. In our study as well as in previous studies, the right lower quadrant or ileocecal region of the mesentery was the region in which enlarged lymph nodes were most often found [10–12].

The long coronal-axis diameters of abdominal lymph nodes, which have not been described in literature before, were often larger than the axial long-axis diameters (68% of the measured lymph nodes), indicating a vertical orientation of the lymph nodes rather than a horizontal orientation. The RECIST and Lugano criteria, which were made for measurements in the axial plane, are not reliably applicable on coronal measurements — the majority of the children (97/152, 64%) in our study population would have had one or more enlarged lymph nodes. Also, when assessing the upper limits of the reference interval, the coronal axes diameters often exceeded the generally used cut-off points of 10 mm for the short axis and 15 mm for the long axis. This is comparable to earlier research on the coronal short and long axis of hilar and mediastinal lymph nodes [14, 15]. Therefore, the coronal axes of abdominal lymph nodes in children should not be used to interpret whether a lymph node is enlarged based on criteria for axial measurements.

There are a few limitations to our study that should be addressed. First, the measurements reported were performed by a medical student with limited experience measuring lymph nodes. To determine the accuracy of the measurements, a sample of 20 CT scans was separately evaluated by a 25-year experienced paediatric radiologist, and a very good interobserver agreement was shown. Second, the study group consisted of children after trauma, which potentially could have influenced the lymph node size. However, it is not likely that the size of lymph nodes would increase substantially in the first hours after trauma. Because our study group consisted of children after high-energy trauma we think this population represents the best possible sample of the healthy population, and after checking the medical records it was confirmed that none of them had a malignancy or infectious disease. Additionally, all measured lymph nodes showed one or multiple aspects indicating benignancy (symmetry, oval shape and fat attenuation). In clinical practice, size measurements alone are, of course, not enough to assess whether a lymph node is pathological, and other factors should be taken into account, as well, for both enlarged lymph nodes and those of normal size.

## Conclusion

Our study shows that the prevalence of abdominal lymph nodes at CT in children is high and that abdominal lymph node size is correlated with age. Despite the smaller body size, the current RECIST and Lugano guidelines for enlarged lymph nodes seem adequate for children in most lymph node stations for the axial measurements. However, for the inguinal station, the criteria do not apply, particularly in adolescents.
